# Preventive Effects of Different Black and Dark Teas on Obesity and Non-Alcoholic Fatty Liver Disease and Modulate Gut Microbiota in High-Fat Diet Fed Mice

**DOI:** 10.3390/foods11213457

**Published:** 2022-10-31

**Authors:** Bangyan Li, Qianqian Mao, Ruogu Xiong, Dandan Zhou, Siyu Huang, Adila Saimaiti, Ao Shang, Min Luo, Hangyu Li, Huabin Li, Sha Li

**Affiliations:** 1 Guangdong Provincial Key Laboratory of Food, Nutrition and Health, Department of Nutrition, School of Public Health, Sun Yat-Sen University, Guangzhou 510080, China; 2 School of Public Health, Shanghai Jiao Tong University School of Medicine, Shanghai 200025, China

**Keywords:** tea, *Camellia sinensis*, black tea, dark tea, non-alcoholic fatty liver disease, obesity, gut microbiota

## Abstract

Non-alcoholic fatty liver disease (NAFLD) has emerged as a leading public health challenge and is closely associated with metabolic syndromes, such as obesity. Intestinal microbiota dysbiosis could play a vital role in the pathogenesis and progression of NAFLD. Tea is the second most popular health drink in the world behind water, and exhibits many health-promoting effects. In this study, the protective effects of different black and dark teas on NAFLD induced by long-term high-fat diet (HFD) exposure and their regulation of gut microbiota were evaluated and explored. The results indicated that supplementation with different black and dark tea extracts could significantly suppress the energy intake, alleviate abnormal accumulation of visceral fat, and prevent obesity, hepatic abnormal lipid deposition and liver steatosis in HFD-fed mice at varying degrees. In addition, Dianhong tea and Liupao tea interventions could significantly decrease the ratio of *Firmicutes* to *Bacteroidetes*, and selenium-enriched black tea and selenium-enriched dark rea supplementation could remarkably reduce the relative abundance of *Actinobacteria* compared to the model group. Moreover, these teas could partly shift the relative abundances of *Allobaculum*, *Roseburia* and *Dubosiella*. Taken together, black teas and dark teas could prevent HFD-induced features of obesity and NAFLD, which might partly be due to the modulation of gut microbiota.

## 1. Introduction

Non-alcoholic fatty liver disease (NAFLD), a predominant contributor of chronic liver disease, has emerged as a leading public health challenge due to its high prevalence, complex pathogenesis, difficulty in diagnosis and lack of authorized therapeutic approaches [1,2]. Mounting evidence has documented that the initiation and progression of NAFLD is also closely related to metabolic disorders including obesity, with an estimated global prevalence of 25–30% for NAFLD and up to 90% in morbidly obese patients [3]. NAFLD could further proceed to steatohepatitis, fibrosis, cirrhosis and hepatocellular carcinoma [4]. Therefore, the prevention and management of NAFLD is crucial. To date, lifestyle modification (health diet and exercise) has proven to be the first-line therapy for NAFLD, but sustaining changes long-term is elusive for many individuals to achieve [5]. Consequently, novel and more practical therapeutic approaches to prevent and manage NAFLD have been urgently required and explored.

To date, accumulating evidence has indicated that diet and intestinal microbiota could be essential factors in the pathogenesis and progression of NAFLD [6,7,8]. Additionally, dietary factors are known to lead to changes in the composition of gut microbiota, generally in the form of variations in the abundance of beneficial species and some specific pathogenic ones [9]. It has demonstrated that gut microbiome dysbiosis could lead to certain adverse effects, such as intestinal permeability dysfunction, elevated endotoxin level in the circulatory system, metabolic endotoxemia and abnormal lipid metabolism, which ultimately induced the obesity and hepatic steatosis in view of the intimate relationship between the liver and gut [10]. On the other hand, considerable research has revealed that prolonged high-fat diet (HFD) exposure could alter gut microbiota composition and lead to gut microbiota dysbiosis [11]. For instance, previous studies reported that an obvious reduction in the relative abundances of *Bacteroidetes*, *Parabacteroides distasonis* and *Ruminococcaceae Oscillospira*, and a significant increase in that of *Firmicutes*, *Allobaculum*, *Lactobacillales* and *Lactobacillus* were observed in the HFD-fed mice, which could influence hepatic steatosis, and the gut microbiota played a preeminent role in the progression of NAFLD [8,12,13]. Furthermore, some foods and their functional components have recently been identified to have the potential ability to modulate the gut microbiota composition, repair the functional intestinal barrier, thereby regulating abnormal lipid metabolism and preventing obesity and NAFLD [14,15,16]. Thus, the gut microbiota was considered as an additional potential target for the development of therapeutic agents or nutritional interventions for NAFLD.

Tea is a popular health drink in the world [17]. Tea has a variety of beneficial effects, such as antiobesity, hepatoprotective, antioxidant and anticancer effects, which are largely attributed to its main functional active components, such as polyphenols [18,19,20]. In addition, previous studies have found that polyphenols from tea are usually poorly absorbed, and that some polyphenols could be utilized by the intestinal microbiota in the large intestine, which might promote the beneficial effects ascribed to their metabolites by gut microbiota [20,21]. For another thing, based on different extents of fermentation and processes, tea is generally categorized into six major groups, including green tea (unfermented), white tea (mildly fermented), yellow tea (lightly fermented), oolong tea (semi-fermented), black tea (deep-fermented) and dark tea (postfermented) [22]. In recent years, mounting attention has been focused on impact of the black and dark teas in modulating intestinal microbiota due to the many microorganisms and their metabolites produced during their fermentation process [23,24]. For instance, a previous animal study suggested that the increased *Firmicutes*/*Bacteroidetes* ratio induced by HFD was significantly restored by the supplementation of Fuzhuan brick tea (a dark tea) [25]. Similarly, another study reported that Fuzhuan brick tea could remarkably increase the abundances of some beneficial bacteria, such as *Clostridiaceae*, *Bacteroidales*, and *Lachnospiraceae*, and reduce the abundances of certain harmful bacteria, including *Ruminococcaceae*, *Peptococcaceae*, *Peptostreptococcaceae*, and *Erysipelotrichaceae* to improve metabolic disorders induced by HFD [24]. Moreover, it has been pointed out that obesity- and diabetes-related symptoms could be alleviated by treatment with Lapsang souchong (a black tea), which is dependent on regulating the gut microbiota composition, altering the relative abundances of *Lactobacillus*, *Lachnospiraceae* and *Roseburia* [26]. However, the relationship between the prevention and management of NAFLD and the modulatory effects of the gut microbiota of deep-fermented black teas and postfermented dark teas is still very limited, which results in the necessity for further investigation. Thus, the objective of the current study is to investigate and understand the effects of supplementation with different black teas and dark teas on HFD-induced NAFLD, and to determine whether gut microbiota is the potential contributor in the prevention and management of NAFLD by these teas.

## 2. Materials and Methods

### 2.1. Preparation of Tea Extracts and Determination of Bioactive Compounds in Teas

The detail information of three black teas and three dark teas obtained from China is displayed in Table 1. Based on our previous report [27], six kinds of teas were separately extracted, filtered, concentrated and lyophilized, after which the tea extracts were finally obtained. Briefly, 10 g of tea was extracted by 100 mL of boiling water in a water bath (Sensin, Shanghai, China) at 98 °C for 10 min. The extraction was repeated three times, and then all extracted solutions were merged and filtered. Subsequently, the solution was concentrated under a vacuum environment using a rotary evaporator (R-501, Cancun, Shanghai, China) and lyophilized by a freeze dryer (Labconco-7752001, Kansas City, MO, USA) to obtain the final tea extract, which was stored at −80 °C for further experimentation. On the other hand, the main bioactive compounds in six tea extracts were analyzed qualitatively and quantitatively by high-performance liquid chromatography (HPLC) methods by a comparison with retention time and UV-visible spectra of the standard compounds from Derick Biotechnology Co., Ltd. (Chengdu, China) according to our previous report [28]. Briefly, the temperature of separation was 35 °C and the flow rate was 1.0 mL/min. In addition, the mobile phases A and B were 0.1% formic acid (*v*/*v*) and methanol, respectively. The elution gradient was carried out as follows: 0 min (2% B), 10 min (17% B), 15 min (19% B), 20 min (22% B), 40 min (47% B), 50 min (50% B), 60 min (58% B), 70 min (2% B), and 75 min (2% B).

### 2.2. Animal and Experimental Study

**Figure 1:**
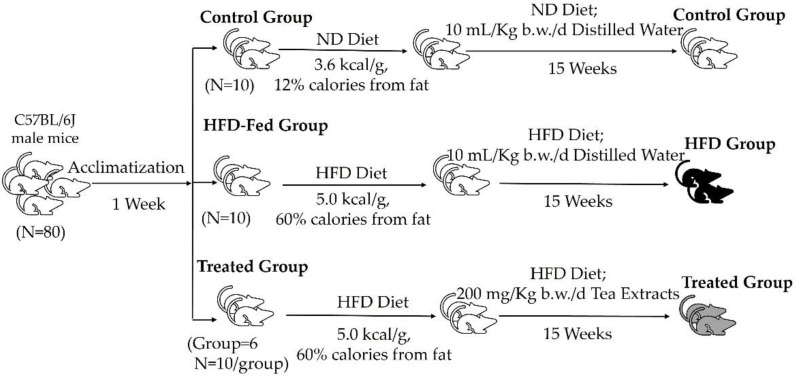
The animal experimental procedure. ND, normal diet; HFD, high-fat diet.

The C57BL/6J male mice were purchased from the Medical Laboratory Animal Centre of Guangdong Province (Guangzhou, China). All mice were housed in specific-pathogen-free condition under a controlled environment with light conditions (12 h light–dark cycle), temperature (22 ± 0.5 °C) and the relative humidity (40–60%) and had libitum access to diet and water. After passing the inspection and quarantine as well as one-week acclimatization, the 8-week-old mice were randomly divided into 8 groups (10 mice per group, 5 mice per cage) based on body weight. As shown in Figure 1, the first group of mice was fed with the same diet (ND, 3.6 kcal/g, 12% calories from fat) as the control group (ND group). In addition, the second group of mice was fed with the same high-fat diet (HFD, 5.0 kcal/g, 60% calories from fat, TP23400) as the model group (HFD group). Moreover, the other groups of mice were maintained on HFD and were supplemented with different tea extracts, including selenium-enriched black tea (BTea1), Dianhong tea (BTea2), Yingde Black tea (BTea3), Fu brick tea (DTea1), Liupao tea (DTea2) and selenium-enriched dark tea (DTea3) at the dose of 200 mg/kg b.w./d for 15 weeks via intragastric gavage according to the previous report [24], while the control group and model group were administered deionized water (10 mL/kg b.w./d). Furthermore, the diets were purchased from TROPHIC Animal Feed High-tech Co., Ltd. (Nantong, China). Importantly, all experimental procedures and protocols involving animals in this research were approved by the School of Public Health of Sun Yat-Sen University in accordance with the “Principles of Care and Use of Laboratory Animals” (approval number: 2019-002; 28 February 2019). During the experimental period, the body weight and the food intake of mice were measured weekly. Finally, all mice in different groups that fasted for 12 h were weighed, anesthetized, and sacrificed, and the fecal sample, blood, liver, epididymal adipose tissue and perirenal adipose tissue were collected.

### 2.3. Measurement of Serum Biochemical Variables

Blood samples were collected and centrifuged at 4000× *g* for 15 min at 4 °C to obtain serum, which was stored at 4 °C for further biochemical testing. Kits for serum triglycerides (TG), total cholesterol (TC), low-density lipoprotein cholesterol (LDL-C), alanine aminotransferase (ALT) and aspartate aminotransferase (AST) were obtained from Roche Diagnostics (Shanghai, China). Additionally, the concentrations of serum TG, TC, and LDL-C, as well as the activities of serum ALT and AST, were measured with an automated biochemistry analyzer (Roche, Mannheim, Germany).

### 2.4. Biochemical Assessment of Liver Tissue

The detection kits of hepatic TG, total protein and malondialdehyde (MDA) were purchased from Apply gen Technologies Inc. (Beijing, China). Liver sample (25 mg) was mixed with 500 µL of lysis buffer and incubated at the condition of 4 °C for 30 min, and then was ground with TissueLyser II Qiagen (QIAGEN^®^, Hilden, Germany). Subsequently, the liver homogenate was heated at 70 °C for 10 min and centrifuged to obtain the supernatant (2000× *g*, 4 °C, for 5 min), which was used for the determination of hepatic TG and total protein concentrations. Moreover, it should be noted that the supernatant used for the measurement of MDA level was obtained by centrifuging the liver homogenate at the condition of 10,000× *g* at 4 °C for 10 min according to the manufacturer’s instructions.

The activity of superoxide dismutase (SOD) and the content of glutathione (GSH) were determined by commercially available kits (Nanjing Jiancheng Bioengineering Institute, Nanjing, China). The frozen liver tissue was mixed with physiological saline solution (0.9%) and homogenized to gain hepatic homogenates, which were centrifuged at the condition of 2500× *g* at 4 °C for 10 min to obtain supernatants for measuring SOD activity and GSH content.

### 2.5. Histopathological Examination

Haematoxylin-eosin (H&E) staining was employed to evaluate the histopathological changes in liver and epididymal adipose tissues. In brief, after mice were sacrificed, liver and epididymal adipose tissues were collected immediately and immersed in 4% paraformaldehyde for two days, and then embedded in paraffin wax. The embedded samples were cut into 5-μm-thick sections and then deparaffinized, rehydrated, and coloured with H&E. Finally, the images of hepatic and epididymal adipose tissues were captured and analyzed with a light microscope (Leica, Solms, Germany). Histological analysis of liver tissue was used for the assessment of the extent of liver damage, such as changes in hepatocyte lipid accumulation and infiltration of inflammatory cells. In addition, histological observation of epididymal adipose tissue was performed to evaluate the size of the adipocytes and determine the degree of obesity.

### 2.6. Bioinformatics Analysis of Intestinal Microbiota

Fresh and uncontaminated fecal samples from each mouse were collected and immediately frozen in liquid nitrogen. The ALFA-SEQ Advanced Stool DNA Kit (Magi gene, Guangdong, China) was used to extract the fecal total microbial genomic DNA. In addition, the integrity and purity of the DNA sample were assessed by the 1% agarose gel electrophoresis. Moreover, the concentration of the extracted DNA was determined by Thermo Nano Drop One (Thermo Fisher, Waltham, MA, USA), as described previously [29].

The V3+V4 high variant region of 16S rRNA was amplified by polymerase chain reaction (PCR) using forward primer 5-ACTCCTACGGGAGGCAGCA-3 and reverse primer 5-GGACTACHVGGGTWTCTAAT-3. Subsequently, the PCR amplification procedure was performed in a thermal cycler with DNA polymerase. Gene Tools Analysis software (Version 4.03.05.0, SynGene, Frederick, MD, USA) was applied to compare the concentrations of PCR products. Afterward, the volume required for each sample was calculated according to the equal mass principle, and the PCR products were mixed. Additionally, the E.Z.N.A. TM gel extraction kit (Omega, Norcross, GA, USA) was implemented to recover the mix PCR products, and the target DNA fragments were eluted with a TE buffer. Finally, the target DNA fragments were built according to the NEBNext^®^ Ultra™ DNALibraryPrepKitforIllumina^®^ standard procedure and were sequenced on a high-throughput Illumina Novaseq 6000 sequencing platform at Guangzhou Magigene Technology Co., Ltd. (Guangzhou, China). The raw image data files obtained from sequencing were converted into raw reads by base calling analysis, and the results were stored in FASTQ file format, containing the sequence information of sequenced reads and its corresponding sequencing quality information.

Quantitative insight into microbial ecology (QIIME) (http://qiime.org/, accessed on 18 January 2021) was implemented to clean all raw data of intestinal microbiota information in stool samples to gain the high-quality clean labels. After that, an operational taxonomic unit (OUT) was generated and clustered as a threshold, with respect to all sequences with 97% identity. Firstly, alpha diversity was utilized to compare the richness (Chao 1’s index) and diversity (Simpson’s index and Shannon’s index) of microbial communities among experimental groups. Secondly, beta-diversity analysis was determined based on principal coordinates (PCoA) analysis using permutational MANOVA (PERMANOVA) analysis (Bray–Curtis distance) to analyze the heterogeneity of microbial community. In addition, the differentially abundant groups of intestinal microbiotas (from phylum to genus) were explored using linear-discriminant analysis effect size analysis (LEfSe) method, and the statistical significance of the differentially abundant groups represented by the clades was shown in accordance with the (LDA) score > 4. Finally, spearman correlation analysis was conducted to analyze the correlation between gut microbiota communities and NAFLD-related properties in R software (Version 3.1.0). Furthermore, *p* values were corrected using Benmaini–Hochberg method.

### 2.7. Statistical Analysis

In this study, all experimental data among groups were analyzed using SPSS 20.0 software (IBM SPSS Statistics, IBM Corp, Somers, NY, USA) and the results were expressed as mean ± standard deviation (SD). Additionally, one-way analysis of variance (ANOVA) and least-significant difference (LSD) tests were used for comparing the statistical differences between the experimental groups. Moreover, GraphPad Prism 8 software (GraphPad software, La Jolla, CA, USA) and R software (Version 3.1.0) were used for drawing graphs and other analyzes. The results of statistical analysis were considered statistically significant when the *p* value was <0.05.

## 3. Results and Discussion

### 3.1. Effects of Tea Extracts on Obesity

**Figure 2:**
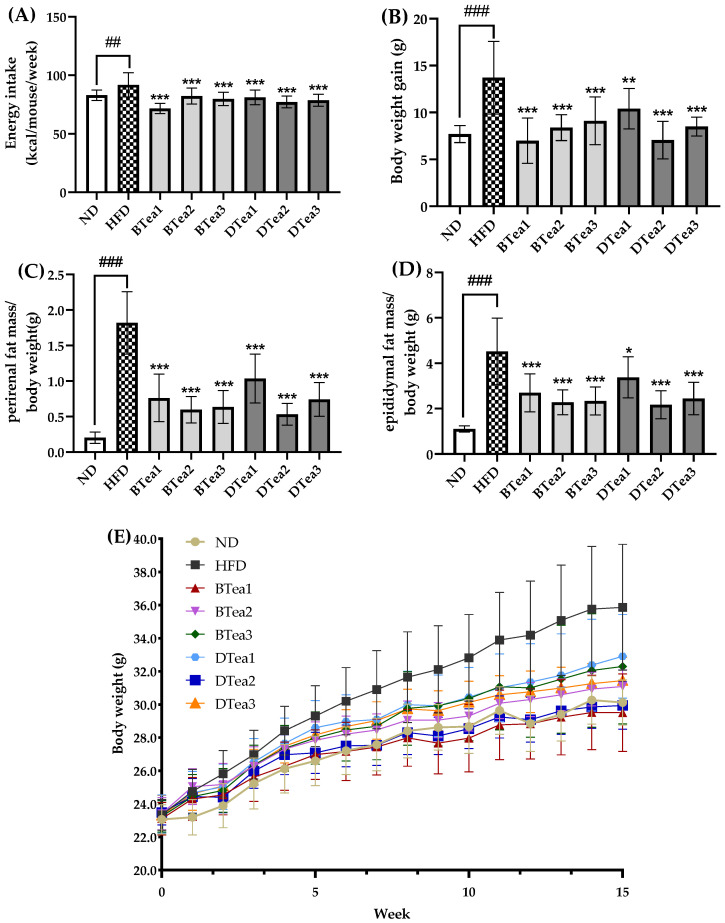
The effects of different black and dark teas on obesity. (**A**) Energy intake, (**B**) body weight gain, (**C**) the ratio of perirenal fat mass to body weight, (**D**) the ratio of epididymal fat mass to body weight, (**E**) and body weight throughout the 15-week intervention. The results are expressed as mean ± standard deviation. ND, the control group (normal diet group); HFD, the model group (high-fat diet group); BTea1, selenium-enriched black tea; BTea2, Dianhong tea; BTea3, Yingde Black tea; DTea1, Fu Brick Tea; DTea2, Liupao tea; DTea3, selenium-enriched dark tea. ## *p* < 0.01 and ### *p* < 0.001f for the HFD group, compared with the ND group; * *p* < 0.05, ** *p* < 0.01, *** *p* < 0.001, the tea extract supplementary groups compared with the HFD group.

To assess the influences of three black teas and three dark teas on the initiation and development of obesity, the male C57BL/6J mice were fed with HFD and supplemented with 200 mg/kg b.w. of these tea extracts. After a 15-weeks intervention, the effects of these tea extracts on weekly energy intake, body weight, perirenal and epididymal fat accumulation in mice exposed to HFD feeding are presented in Figure 2.

When compared to the control group, the final body weight of the model group increased by 19.09%, which indicated that the mice in HFD group had developed obesity. Accumulating evidence has demonstrated that chronic and excessive HFD exposure caused weight gain and abnormal lipid accumulation, resulting in obesity [30]. In additionally, the weekly energy intake, as well as body weight gain in HFD groups, were significantly increased to 1.11-fold (91.86 ± 10.30 vs. 82.89 ± 4.46) (*p* < 0.01) and 1.78-fold (13.71 ± 3.87 vs. 7.70 ± 0.91) (*p* < 0.001) the ND group, respectively. As displayed in Figure 2A,B, all tea extract supplementation showed a significant inhibitory effect on the weekly energy consumption and the body weight gain in comparison with the HFD group, suggesting that these black and dark teas could play a role in inhibiting obesity. It has been proved that weight loss is usually accompanied by a reduction in energy intake [31]. In this study, the reduction in energy intake was generally consistent with the suppression of body weight gain, and the results were consistent with previous reports [25]. It has revealed that the body weight gain caused by HFD exposure was significantly attenuated by supplementation with a Fuzhuan brick tea (a type of dark tea) extract at a dose of 400 mg/kg b.w. for 8 weeks [25]. Moreover, a previous study revealed that epigallocatechin gallate (EGCG) in tea promoted the reduction of food intake and body weight via appetite suppression [32]. Correspondingly, we found that the greatest decrease in body weight gain, of 48.90% and 48.18%, was observed in the selenium-rich black tea (BTea1) and Liubao tea (DTea2) supplementation groups, respectively, which were coincided with the greatest reduction in their weekly energy intake. Therefore, the antiobesity effects of different black and dark teas might be partly attributed to their effects of reducing energy intake.

On the other hand, the ratios of perirenal and epididymal fat mass to body weight were employed to assess the effects of different black and dark teas on visceral fat, which are presented in Figure 2C,D. The percentage changes in the ratios of perirenal and epididymal fat mass to body weight were 7.69-fold (1.82 ± 0.44 vs. 0.20 ± 0.08) (*p* < 0.001) and 3.11-fold (4.52 ± 1.47 vs. 1.10 ± 0.14) (*p* < 0.001) higher in mice in the HFD group compared with the ND group, respectively. However, the elevations in the ratios of perirenal and epididymal fat mass to body weight caused by HFD were significantly inhibited at varying degrees by supplementation with all tea extracts. It has been demonstrated that Fuzhuan brick tea and black tea significantly decreased body weight gain and abnormal fat accumulation in mice fed with HFD [33]. Moreover, previous studies have pointed out that caffeine, tea polyphenols and tea polysaccharides in tea could prevent the initiation and progression of obesity in HFD-exposed rats [34].

Taken together, supplementation with different black tea and dark tea extracts could attenuate features of obesity, including inhibition of weekly energy intake and body weight gain and reduction in the ratios of perirenal and epididymal fat mass to body weight. In addition, the antiobesity effects of these tea extracts might be partly associated with their effects on the energy intake reduction.

### 3.2. Observation of Histopathological Changes in Adipose Tissues

**Figure 3:**
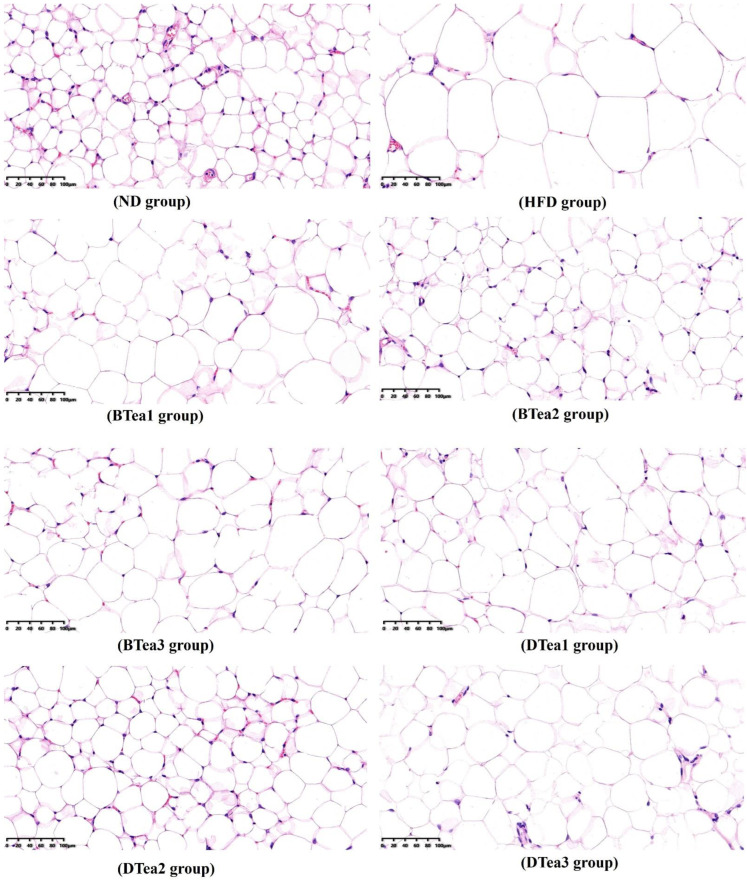
The histopathological changes in epididymal adipose tissue stained with hematoxylin and eosin (H&E) for all groups (magnification: 200, scale bar: 100 µm). ND, the control group (normal diet group); HFD, the model group (high-fat diet group); BTea1, selenium-enriched black tea; BTea2, Dianhong tea; BTea3, Yingde black tea; DTea1, Fu brick tea; DTea2, Liupao tea; DTea3, selenium-enriched dark tea.

The preventive effect of supplementation with different black and dark tea extracts on obesity induced by prolonged HFD exposure were further validated by the histopathological evaluation of epididymal tissue by H&E staining. As displayed in Figure 3, the epididymal adipose tissue morphology in the HFD group was abnormal, which was mainly manifested by the incredible expansion of adipocytes in size and their abnormal irregularity in shape. However, compared with the model group, the adipocytes of all tea extract supplementary groups presented fewer pathological changes, including being much smaller in size and having regular shapes, especially Dianhong tea (BTea2) and Liupao tea (DTea2). The results of the study indicated that these tea extracts had a stronger preventive effect on abnormal visceral fat accumulation. A study revealed that abnormal visceral adipose tissues, such as epididymal and perirenal fats, could store large amounts of TG; when visceral adipose tissue was lipolyzed, the level of free fatty acids in hepatocytes increased, which was a strong predictor of the occurrence and development of NAFLD [35]. Several studies indicated that people with a higher percentage of visceral fat were more likely to develop NAFLD when they had similar body weight [36]. In addition, a previous study reported that black tea supplementation lowered the mean size of epididymal adipocyte and alleviated lipid metabolism disorder and fatty liver, as well as impaired hepatic function in mice fed with a HFD [26].

In summary, supplementation with these black and dark tea extracts could alleviate abnormal accumulation of visceral fat, especially Dianhong Tea (BTea2) and Liupao Tea (DTea2) and might be associated with the prevention and control of lipid metabolism disorder and NAFLD as well.

### 3.3. Effects of Tea Extracts on Features Related to NAFLD

**Figure 4:**
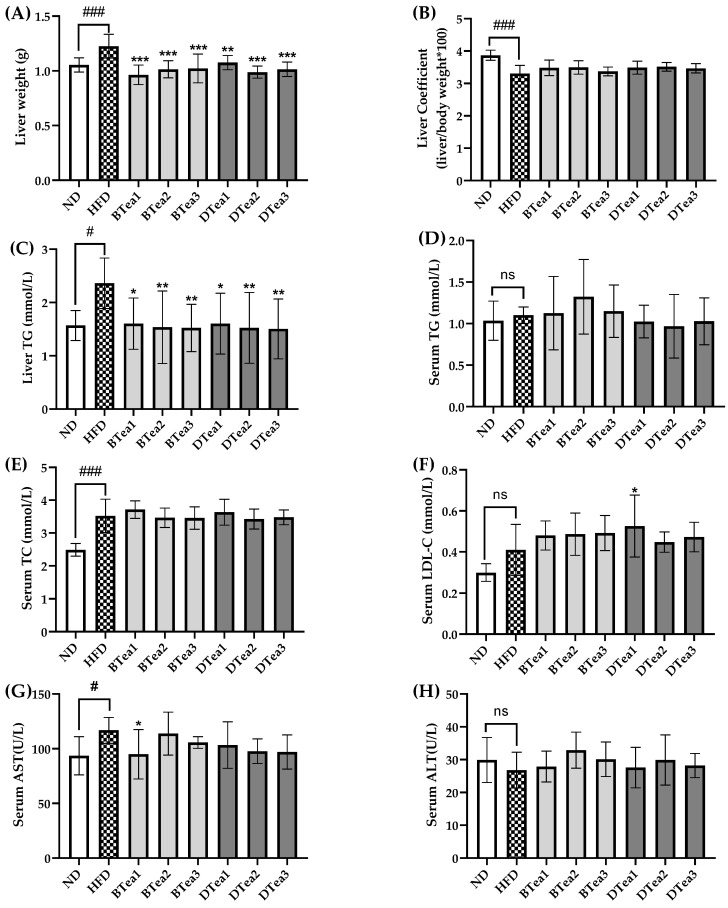
The effects of different black and dark teas on the features related to NAFLD. (**A**) Liver weight; (**B**) liver coefficient; (**C**) liver triacylglycerol level (Liver TG); (**D**) serum triacylglycerol level (Serum TG); (**E**) serum total cholesterol level (Serum TC); (**F**) serum low-density lipoprotein cholesterol level (Serum LDL-C); (**G**) serum aspartate transaminase (Serum AST); (**H**) serum alanine aminotransferase (Serum ALT). The results are expressed as mean ± standard deviation. ND, the control group (normal diet group); HFD, the model group (high-fat diet group); BTea1, selenium-enriched black tea; BTea2, Dianhong tea; BTea3, Yingde black tea; DTea1, Fu brick tea; DTea2, Liupao tea; DTea3, selenium-enriched dark tea. # *p* < 0.05, ### *p* < 0.001, the HFD group compared with the ND group; * *p* < 0.05, ** *p* < 0.01, *** *p* < 0.001, the tea extract supplementary groups compared with the HFD group. ns, no statistically significant differences (*p* > 0.05) between the model group and the control group.

As presented in Figure 4A–C, the HFD group showed a significant increase in liver weight (*p* < 0.001) and hepatic TG concentration (*p* < 0.05), and an obvious reduction in liver coefficient (*p* < 0.001) in comparison with the ND group. However, the administration of all six teas substantially inhibited the elevated liver weight and hepatic TG level induced by the HFD-feeding. The results indicated that these black and dark teas could attenuate the abnormal lipid accumulation in liver caused by the prolonged HFD exposure, which agreed with the effects of other tea type (such as green tea) reported by the previous study [37]. In recent years, a growing number of studies have identified tea supplementation as a potentially implementable nutritional strategy to protect against the hepatic impairment and abnormal lipid metabolism in the liver [24,38]. In this study, although all tea extract supplementary groups showed a trend of elevated liver coefficient compared with the model group, there was no significant statistical difference as shown in Figure 4B.

The impacts of these teas on serum biochemical determinations, including TG, TC and LDL-C concentrations, are displayed in Figure 4D,F. It was noticed that HFD resulted in significant elevation in serum TC level (Figure 4E), while no significant differences were observed in serum TG and LDL-C levels between the ND group and HFD group. It is well known that lipid metabolism disorder is one of the most prominent manifestations of NAFLD, which could be presented as hypertriglyceridemia, hyperlipidemia and hypercholesterolemia [39]. In this study, most black and dark tea extracts supplementation did not significantly affect the levels of serum TG, TC and LDL-C compared with the HFD group. However, the elevated serum LDL-C level induced by the HFD continued to increase again after supplementation with Fu Brick Tea (DTea1) for 15 weeks. A previous study reported that the levels of serum TC and LDL-C were significantly decreased, but the serum TG was not affected by the treatment with Fuzhuan brick tea at a dose of 400 mg/kg b.w. for 8 weeks [25]. Another research revealed that HFD-induced higher concentrations of serum TC, TG and LDL-C were suppressed by Pu-erh Tea (a dark tea) treatment for 8 weeks [38]. Therefore, the effects of tea on serum lipid levels were controversial according to different studies, which might be due to the differences in dosage and duration of tea treatment, and more studies are needed to explore this in the future. Overall, the results of our study suggested that most black and dark tea extracts had limited effect on blood lipid levels.

Elevated serum concentrations of ALT and AST have been found to be markers of liver injury. As can be seen from Figure 4G,H, the HFD group exhibited an obvious increase in serum AST activity (*p* < 0.05) compared to the ND group, indicating liver damage induced by long-term HFD exposure. The increase in AST activity in the model group was significantly inhibited by selenium-enriched black tea (BTea1) supplementation, suggesting its preventive effect on hepatotoxicity. However, there was no significant difference in serum ALT activity among the ND, HFD, and all tea extract supplementary groups.

In summary, these black and dark tea extracts intervention could reduce abnormal accumulation of hepatic lipids and prevent the onset and development of fatty liver, but these black and dark tea extracts interventions have limited effects on regulating blood lipid levels and alleviating hepatotoxicity.

### 3.4. Histopathological Evaluation for Hepatic Tissues

**Figure 5:**
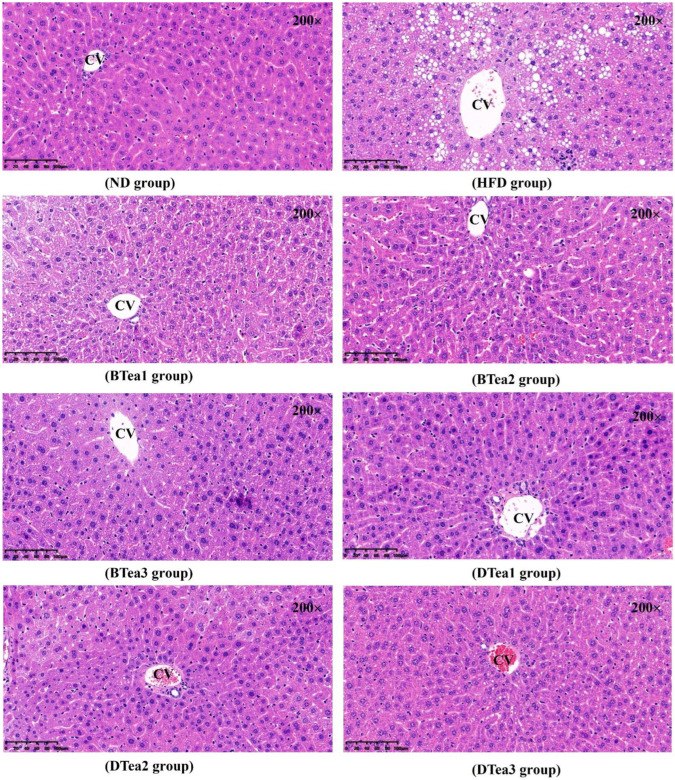
The histopathologic evaluations in hepatic tissue stained with haematoxylin and eosin (H&E) for all groups (magnification: 200, scale bar: 100 µm). ND, the control group (normal diet group); HFD, the model group (high-fat diet group); BTea1, selenium-enriched black tea; BTea2, Dianhong tea; BTea3, Yingde black tea; DTea1, Fu brick tea; DTea2, Liupao tea; DTea3, selenium-enriched dark tea.

To further confirm the preventive effect of different black and dark tea extracts on NAFLD caused by chronic HFD consumption, H&E staining was employed to observe the hepatic histopathological morphologies of the hepatic tissue (Figure 5). No significant pathological damage was observed in the liver tissue of the ND group, whereas the liver tissue of the HFD group presented a disorder of hepatocytes arrangement, deposits of excessive medium and small lipid droplets in the parenchyma cells, and inflammatory infiltrate, indicating the emergence of NAFLD. The supplementation with these different black and dark teas could obviously reduce lipid droplets in hepatocytes and attenuate the liver damage as well as inflammatory infiltrate, which were consistent with their inhibitory effects on HFD feeding-induced increased liver weight and hepatic TG level (see previous section).

### 3.5. Effects of Tea Extracts on Liver Oxidative Damage

**Figure 6:**
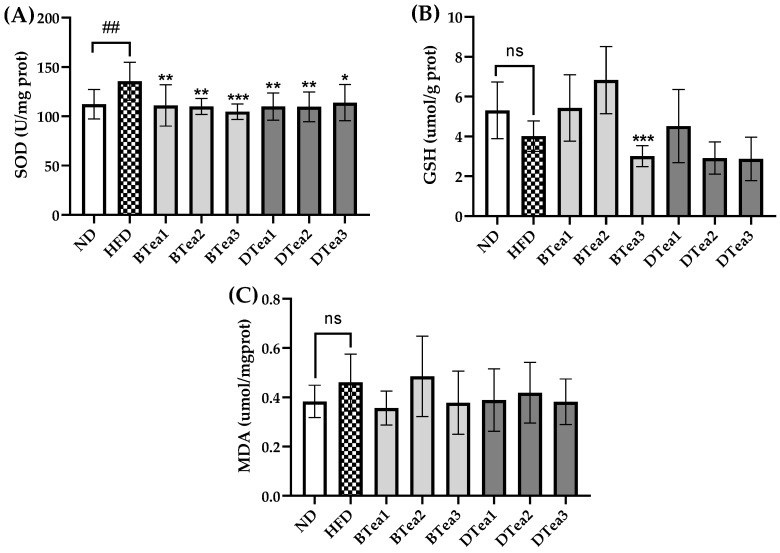
The effects of different black and dark teas on liver oxidative injury. (**A**) superoxide dismutase (SOD); (**B**) glutathione (GSH); (**C**) malondialdehyde (MDA). The results are expressed as mean ± standard deviation. ND, the control group (normal diet group); HFD, the model group (high-fat diet group); BTea1, selenium-enriched black tea; BTea2, Dianhong tea; BTea3, Yingde black tea; DTea1, Fu brick tea; DTea2, Liupao tea; DTea3, selenium-enriched dark tea. ## *p* < 0.01, the HFD group compared with the ND group; * *p* < 0.05, ** *p* < 0.01, *** *p* < 0.001, the tea extract supplementary groups compared with the HFD group. ns, no statistically significant differences (*p* > 0.05) between the model group and the control group.

It has been demonstrated that the liver possesses a powerful antioxidant system, containing such substances as non-enzymatic antioxidants (GSH) and antioxidant enzymes (SOD), to defend against the production of excessive ROS [40]. However, the stable antioxidative system in vivo could be disrupted by the accumulation and oxidation of excessive fatty acids induced by HFD exposure [41], which could destroy the structure and functions of hepatocytes and eventually promote the initiation and progression of NAFLD [42]. In addition, MDA is one of the biomarkers of lipid peroxidation, and changes in its level could suggest the severity of hepatic lipid peroxidation and the extent of liver injury. As shown in Figure 6, the MDA level in the HFD group presented an increasing trend and the GSH content showed a decreasing trend compared with the ND group, which indicated that oxidative stress was likely to occur in the model group. Most tea extract supplementation did not affect MDA and GSH contents, but the GSH level was further decreased by the administration of Yingde black tea (BTea3) compared with the model group. In the present study, long-term HFD intake resulted in elevated SOD activity compared with the control group, while supplementation with all tea extracts significantly reduced the elevated SOD activity. The results of the present study were inconsistent with the previous reports [43], which reported that the administration of green tea extract for 6 weeks suppressed ROS production and reduced lipid peroxidation in the non-alcoholic steatohepatitis model. This could be due to the long-term supplementation of tea extracts, resulting in the antioxidants level being too high, which showed pro-oxidant activity in vivo. This was similar to our previous findings, which indicated that most tea extracts were strong antioxidants in vitro but presented pro-oxidant activity in vivo [27,28].

### 3.6. Effects of Tea Extracts on the Diversity and Structure of Gut Microbiota

An increasing amount of evidence indicated that the intestinal microbiota dysbiosis was highly linked with the pathogenesis of NAFLD [44,45]. In addition, it has been reported that diets exhibit a dominant role in shaping the diversity, structure and composition of gut microbiota [46]. In the present work, pyrosequencing of high variant region V3 + V4 based on bacterial 16S rRNA genes was conducted to determine the influence of HFD, deep-fermented black teas and postfermented dark teas on the diversity and structure of gut microbiota community. As presented in Table 2, the richness and diversity of the gut microbiota were analyzed based on alpha-diversity analysis, including Chao 1, Simpson and Shannon, with Chao 1 reflecting community richness, and Simpson and Shannon representing community diversity. The results suggested that the differences in gut microbiota richness and diversity between HFD group and ND group were insignificant, although there was a trend towards decreasing gut microbiota diversity in the HFD group. However, compared with HFD group, selenium-enriched black tea (BTea1) obviously increased the microbiota richness. In addition, most of other tea extracts treatment non-significantly influenced the gut microbiota richness and diversity. Moreover, we found that Dianhong Tea (BTea2) and Liupao Tea (DTea2) interventions could ameliorate the microbiota phylogenetic richness and diversity, just as Fu trick tea (DTea1) significantly decreased that variety of richness in comparison with the selenium-enriched black tea (BTea1) group.

**Figure 7:**
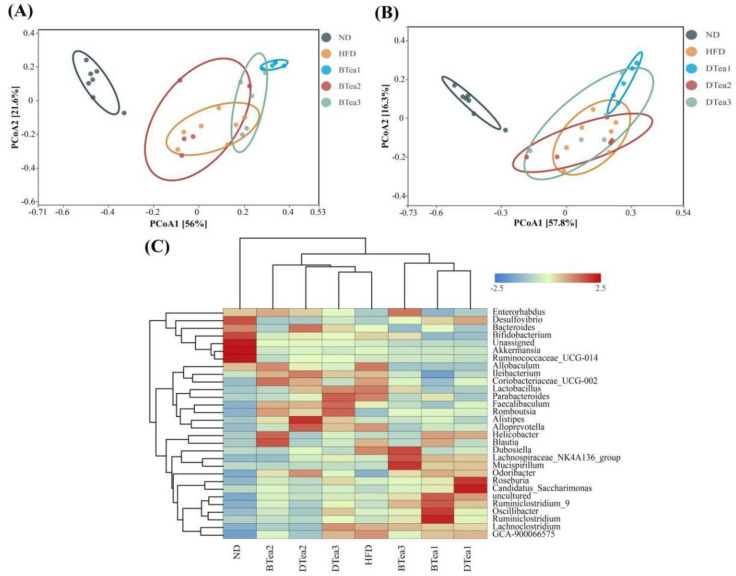
The effects of different black and dark teas on the gut microbiota structure. (**A**) PCoA analysis among ND, HFD and black teas groups; (**B**) PCoA analysis among ND, HFD and dark teas groups; (**C**) species abundance cluster analysis at the genus level. ND, the control group (normal diet group); HFD, the model group (high-fat diet group); BTea1, selenium-enriched black tea; BTea2, Dianhong tea; BTea3, Yingde black tea; DTea1, Fu brick tea; DTea2, Liupao tea; DTea3, selenium-enriched dark tea.

The beta-diversity analysis and species abundance clustering analysis at genus level were performed to investigate the similarity and differences between gut microbiota communities, in structure and composition, among different groups. As displayed in Figure 7A,B as well as Table 3, beta-diversity analysis based on permutational MANOVA (PERMANOVA) analysis using Bray–Curtis distance for principal coordinates (PCoA) analysis reported that significant separation was noted for HFD and ND groups (PERMANOVA, *p* = 0.001), suggesting that the microbial structure and composition were dramatically disturbed by HFD exposure compared with the ND group. In addition, the results showed that selenium-enriched black tea (BTea1) (PERMANOVA, *p* = 0.003), Yingde black tea (BTea3) (PERMANOVA, *p* = 0.014) and Fu brick tea (DTea1) (PERMANOVA, *p* = 0.002) interventions significantly separated from those of the HFD group, which indicated these teas supplementation could have substantial effects on the HFD-disrupted gut microbiota. Moreover, as seen from Figure 7A, PCoA1 accounted for 56.0% of the total variance, which was higher than PCoA2, which accounted for 21.6% of the total variance. This illustrated that selenium-rich black tea (BTea1) and Yingde black tea (BTea3) could suppress HFD-induced changes in gut microbiota composition, indicating in turn that PCoA1 could reflect the main effects of diet on gut microbiota composition. Nevertheless, the influences of microbiota structure caused by HFD exposure could not be inhibited by the supplementation of Dianhong tea (BTea2), Liupao tea (DTea2) and selenium-enriched dark tea (DTea3), because the gut microbiota structure and composition in these tea supplementary groups were insignificantly changed compared with the HFD group (PERMANOVA, *p* > 0.05). Furthermore, the differences in gut microbiota composition among the black tea (BTea2), Liubao tea (DTea2) and selenium-rich black tea (DTea3) groups were insignificant (PERMANOVA, *p* > 0.05), suggesting that these teas exhibited limited effects on the HFD-induced differences in gut microbiota composition. On the other hand, a significant separation was also observed in the results of species abundance cluster analysis at genus level (Figure 7C) between HFD and ND groups, which was consistent with the results of PCoA analysis. In addition, our present work revealed that selenium-enriched black tea (BTea1), Yingde black tea (BTea3) and Fu brick tea (DTea1) were also in the same cluster and substantially distant from the model group, while the microbial community composition was similar among Dianhong tea (BTea2), Liupao tea (DTea2) and selenium-enriched dark tea (DTea3) and HFD groups.

### 3.7. Effects of Tea Extracts on the Composition of Gut Microbiota

**Figure 8:**
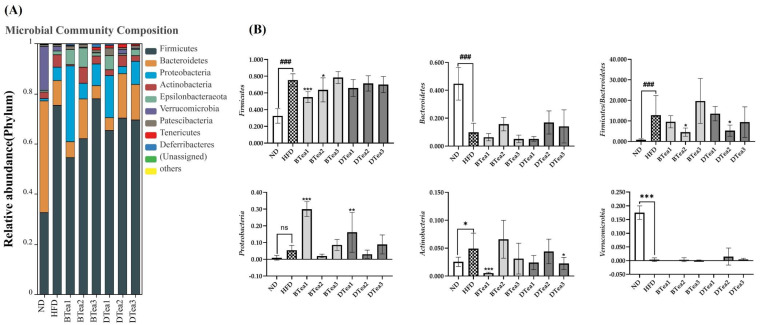

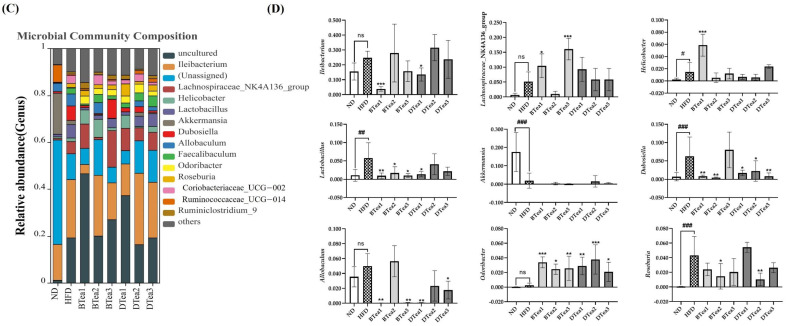
The effects of different black and dark teas on the gut microbiota composition at the phylum and genus levels. (**A**) Relative abundance of the gut microbial profile at the phylum level among all groups; (**B**) relative abundance of *Firmicutes*, *Bacteroidetes*, the ratio of *Firmicutes* and *Bacteroidetes*, *Proteobacteria*, *Actinobacteria* and *Verrucomicrobia* among all groups; (**C**) relative abundance of the gut microbial profile at the genus level among all groups; (**D**) relative abundance of *Ileibacterium*, *Lachnospiraceae*_NK4A136_group, *Helicobacter*, *Lactobacillus*, *Akkermansia*, *Dubosiella*, *Allobaculum*, *Odoribacter* and *Roseburia* among all groups. The results are expressed as mean ± standard deviation. ND, the control group (normal diet group); HFD, the model group (high-fat diet group); BTea1, selenium-enriched black tea; BTea2, Dianhong tea; BTea3, Yingde black tea; DTea1, Fu brick tea; DTea2, Liupao Tea; DTea3, selenium-enriched dark tea. # *p* < 0.05, ## *p* < 0.01, ### *p* < 0.001, the HFD group compared with the ND group; * *p* < 0.05, ** *p* < 0.01, *** *p* < 0.001, the tea extract supplementary groups compared with the HFD group. ns, no statistically significant differences (*p* > 0.05) between the model group and the control group.

Gut microbiota composition analysis was conducted to analyze the specific changes in the gut microbiota composition at the phylum and genus levels as presented in Figure 8A,B. It has been demonstrated that the gut microbiota structure and composition of mice are predominantly dominated by the *Firmicutes* and *Bacteroidetes* at the phylum level, which is in line with our results in the present study [47,48]. At the phylum level, the HFD feeding induced significant elevation in the relative abundance of *Firmicutes* (*p* < 0.001) and obvious reduction in that of *Bacteroidetes* (*p* < 0.001). Thus, the results indicated that the gut microbial community structure and composition could be dramatically changed by prolonged HFD exposure, with increased ratio of *Firmicutes* to *Bacteroidetes* in comparison with the control group (*p* < 0.001), which was consistent with the previous reports [24,49]. For another thing, previous studies have demonstrated that the ratio of *Firmicutes* to *Bacteroidetes* can be reduced by the treatments of polysaccharides, polyphenols, and other functional components, which contribute to inhibit the HFD-induced body weight gain [48,50]. In the current work, Dianhong tea (BTea2) and Liupao tea (DTea2) interventions could significantly inhibit the elevation of *Firmicutes* to *Bacteroidetes* ratio, gradually approaching the level of ND group, and selenium-enriched black tea (BTea1) and Dianhong tea (BTea2) supplementation could markedly attenuate the increase in the relative abundance of *Firmicutes*. In addition, an increasing trend in the relative abundance of *Bacteroidetes* was observed in Dianhong tea (BTea2) and Liupao tea (DTea2) and selenium-enriched dark tea (DTea3) supplementary groups, but there was no significant difference. It has been demonstrated that *Bacteroidetes* level increased and the ratio of *Firmicutes* to *Bacteroidetes* decreased when body weight was reduced, which might be responsive to calorie intake [6]. Thus, our results indicated that Dianhong tea (BTea2) and Liupao tea (DTea2) and selenium-enriched dark tea (DTea3) could alleviate HFD-induced obesity by reducing the acquisition of energy and nutrient from diet through modulating the gut microbiota. Apart from the *Firmicutes* and *Bacteroidetes* at the phylum level, *Proteobacteria*, *Actinobacteria*, *Epsilonbacteraeota*, *Verrucomicrobia*, *Patescibacteria*, *Tenericutes* and *Deferribacteres* were the most abundant bacteria among all groups with relative abundance > 0.01%. Our present work found that long-term HFD feeding significantly increased the relative abundance of *Actinobacteria* (*p* < 0.05) and decreased that of *Verrucomicrobi* (*p* < 0.001), but showed a limited effect on that of *Proteobacteria, Epsilonbacteraeota*, *Patescibacteria*, *Tenericutes* and *Deferribacteres* (*p* > 0.05). Compared with the HFD group, selenium-enriched black tea (BTea1) and selenium-enriched dark tea (DTea3) treatments could significantly reduce the relative abundance of *Actinobacteria*, which was considered as health-inhibiting specie [51,52]. However, the supplementation of selenium-enriched black tea (BTea1) and Fu brick tea (DTea1) could further significantly enhance the elevation of *Proteobacteria* disturbed by HFD feeding compared with the model group, which was strongly associated with inflammation [53].

Previous study has demonstrated that the functions of bacteria and their roles in metabolic diseases were strain-specific [54]. It is essential to determine the changes in gut microbiota composition at the genus level following different black and dark tea treatments, as the different bacterial genus of the same phylum respond differently to the same environmental stresses (such as HFD feeding) [55,56]. Thus, genus-level bacteria with relative abundance above 0.01% and the top 15 in relative abundance were analyzed to identify specific bacterial phylotypes, which were altered by the interventions of HFD, different black and dark tea extracts based on the community composition analysis as displayed in Figure 8C,D. In this work, significant elevations in *Helicobacter*, *Lactobacillus*, *Dubosiella*, *Faecalibaculum*, *Roseburia*, *Coriobacteriaceae_UCG-002* and obvious decreases in *Akkermansia* and *Ruminococcaceae*_UCG-014 after supplementation with HFD were observed compared with ND group. Compared with the control group, prolonged HFD feeding showed a limited effects on the relative abundances of *Ileibacterium*, *Lachnospiraceae*_NK4A136_group, *Allobaculum* and *Odoribacter* (*p* > 0.05). It has been reported that *Allobaculum* was involved in the initiation and pathogenesis of NAFLD [57]. Our results found that the relative abundance of *Allobaculum* in the HFD group showed an increasing trend, but that the intervention of selenium-enriched black tea (BTea1), Yingde black tea (BTea3), Fu brick tea (DTea1) and selenium-enriched dark tea (DTea3) significantly inhibited the elevated trend. Interestingly, interventions of selenium-enriched black tea (BTea1), Dianhong tea (BTea2), Yingde black tea (BTea3) and Fu brick tea (DTea1) attenuated the increased relative abundance of *Lactobacillus* induced by HFD feeding. Previous studies indicated that *Lactobacillus* has been shown to be endogenously proinflammatory mediator [58,59], although it was thought to be an anti-inflammatory probiotic [60,61]. In addition, it has been reported that the pregnane X receptor (PXR), a xenobiotic-sensing nuclear receptor, was necessary in promoting HFD-mediated increases in *Lactobacillus*, which was related to body weight gain [62]. Hence, the PXR-dependent increase in *Lactobacillus* has been considered as an indicator of intestinal-derived inflammation. Moreover, long-term HFD feeding enhanced the relative abundance of *Dubosiella*, and most teas in addition to Yingde Black Tea (BTea3) supplementation could significantly ameliorate this increase to a level similar to the ND group. Besides, the elevation in the relative abundance of *Roseburia* induced by HFD could be markedly inhibited by Dianhong Tea (BTea2) and Liupao Tea (DTea2) supplementation. Furthermore, all tea extract interventions could dramatically increase the relative abundance of *Odoribacter* compared with the HFD group.

**Figure 9:**
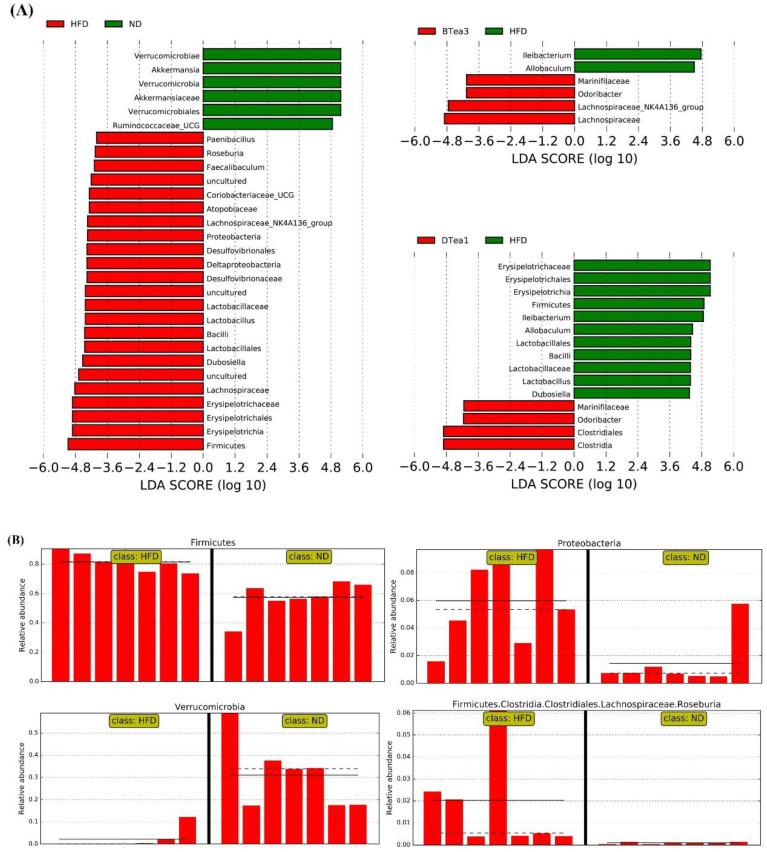
The key differentially abundant taxa of gut microbiota (from phylum, class, order, family to genus levels, respectively) in response to HFD in combination with different black and dark teas treatment. (**A**) LEfSe analysis showed the most divergent abundant taxa. Linear discriminant analysis (LDA) score > 4 is displayed; HFD (red) + ND (green); BTea3 (red) + HFD (green); DTea1 (red) + HFD (green). (**B**) the relative abundance of Phylum of *Firmicutes*, *Proteobacteria* and *Verrucomicrobia* and genus of *Roseburia* between ND group and HFD group from the LEfSe analysis. ND, the control group (normal diet group); HFD, the model group (high-fat diet group); BTea1, selenium-enriched black tea; BTea2, Dianhong tea; BTea3, Yingde black tea; DTea1, Fu brick tea; DTea2, Liupao tea; DTea3, selenium-enriched dark tea.

Linear discriminant analysis (LDA) effect size (LEfSe) analysis was also performed to investigate the differential enrichment in the intestinal microbiota among the control, HFD and all tea extracts supplementary groups ranging from phylum to genus levels. The taxonomic branching plot was produced by using LEfSe analysis of 16S rRNA sequences as shown in Figure 9A, with the bars indicating the effect size for each taxon between the HFD group and ND, Yingde black tea (BTea3) and Fu brick tea (DTea1) groups, respectively. In addition, certain strains including *Firmicutes*, *Proteobacteria, Verrucomicrobia*, and *Roseburia,* that significantly differed between the HFD and control groups, have been displayed in the significantly different strain content graph (Figure 9B). Different enrichment of specific bacteria was observed in both histograms and differential strain content plots, with HFD resulting in a significant alteration in the intestinal microbiota. Compared with the ND group, the *Firmicutes* phylum, *Erysipelotrichia* class, *Erysipelotrichales* order, *Erysipelotrichaceae* family, *Faecalibaculum* genus, *Dubosiella* genus, *Lachnospiraceae* family, *Roseburia* genus, *Bacilli* class, *Lactobacillales* order, *Lactobacillaceae* family, *Lactobacillus* genus, *Proteobacteria* phylum, *Deltaproteobacteria* class, *Desulfovibrionales* order, and *Desulfovibrionaceae* family were enriched in HFD group. However, the supplementation of different black teas and dark teas could partly reverse the shift in gut microbiota induced by HFD feeding. In summary, the intervention of different black and dark tea extracts could effectively attenuate HFD-induced NAFLD through modifying the gut microbiota communities at varying degrees.

### 3.8. The Correlation Analysis between Gut Microbiota and NAFLD Phenotypes

**Figure 10:**
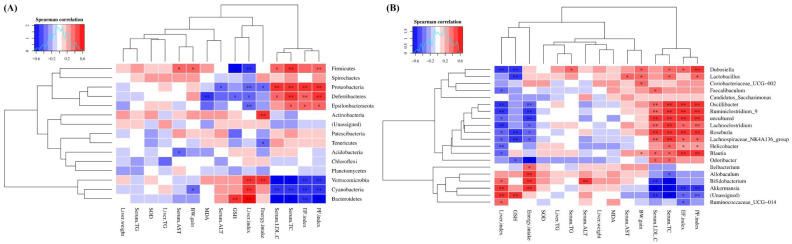
Correlations between the relative abundance of gut microbiota at phylum (**A**) and genus (**B**) levels and NAFLD phenotypes induced by long-term HFD exposure. The NAFLD phenotypes included obesity characteristics, liver damage parameters and liver oxidative injuriers. Asterisk (*) indicates that the correlation between NAFLD phenotypes and the relative abundances of gut microbiota was statistically significant (* FDR < 0.05, ** FDR < 0.01). BW, body weight; EF index, the ratio of epididymal fat mass to body weight; PF index, the ratio of perirenal fat mass to body weight.

In the present study, the relationship between gut microbiota communities at phylum (top 15) and genus (top 20) levels and NAFLD phenotypes induced by long-term HFD exposure was analyzed based on Spearman’s correlation analysis, which is shown in Figure 10A,B, respectively. The link between these major bacteria and obesity characteristics, including energy intake, body weight (BW) gain, epididymal fat (EF) index and perirenal fat (PF) index, was evaluated. At the phylum level, the results indicated that *Firmicutes*, *Proteobacteria*, *Deferribacteres* and *Epsilonbacteraeota* were positively correlated with the EF index and PF index, while the *Bacteroidetes*, *Verrucomicrobia* and *Cyanobacteria* were negatively correlated with these parameters. In addition, *Actinobacteria* and *Verrucomicrobia* showed significant positive correlation with the energy intake, while the *Proteobacteria* and *Tenericutes* presented marked negative association with it. Moreover, *Firmicutes* was positively associated with the BW gain, while *Cyanobacteria* was negatively correlated with it. At the genus level, these bacteria, including *Lachnospiraceae*_NK4A136_group, *Roseburia*, *Lachnoclostridium*, and *Ruminiclostridium*_9, *Oscillibacter* showed significant positive correlations with EF index and PF index, but remarkable negative relationship with energy intake. Besides, the EF index and PF index were negatively related to *Akkermansia.* Furthermore, we found that energy intake was negatively correlated with *Odoribacter* and showed a positive association with *Allobaculum*, *Ileibacterium, Bifidobacterium* and *Akkermansia*. The *Lactobacillus*, *Dubosiella, Coriobacteriaceae*_UCG-002 and *Blautia* were positively correlated with the BW gain.

Further analysis was conducted to evaluate the correlation between gut microbiota and features related to NAFLD, including hepatic steatosis (liver index and liver TG content), serum biochemical indicators of lipid metabolism (serum TG, TC and LDL-C) and serum aminotransferase (AST and ALT) activities. We found that *Firmicutes*, *Proteobacteria* and *Deferribacteres* were positively correlated with Serum TC and LDL-C levels, while they were negatively associated with liver index. In addition, *Bacteroidetes*, *Verrucomicrobia*, and *Cyanobacteria* showed significant negative correlations with Serum TC as well as serum LDL-C levels, while presented dramatical positive association with the liver index. Meanwhile, a negative relationship was observed between the activity of serum ALT and the abundances of *Proteobacteria*. Moreover, the serum AST activity was negatively related to *Acidobacteria*, and was positively associated with *Firmicutes.* The contents of serum TC and LDL-C were positively associated with certain bacteria at the genus level, including *Dubosiella, Lactobacillus, Faecalibaculum, Oscillibacter*, *Ruminiclostridium*_9, *Lachnoclostridium*, *Roseburia*, *Lachnospiraceae*_NK4A136_group, *Helicobacter*, *Blautia* and *Odoribacter*, most of which were also positively related to the liver index. On the other hand, the serum TC and LDL-C levels were negatively correlated to the abundances of *Bifidobacterium* and *Akkermansia*, which were positively associated with liver index. Additionally, the serum TG content was positively correlated to *Dubosiella.* Furthermore, serum aminotransferase (AST and ALT) activities showed marked positive correlations with *Bifidobacterium* and *Lactobacillus*.

The relationship between these gut microbiota and liver oxidative damage, including antioxidant capacity (SOD and GSH) and lipid peroxidation (MDA) levels, has also been investigated. A strong negative correlation was observed between *Deferribacteres* and *Firmicutes* abundances and GSH content, which was positively associated with *Bacteroidetes*. In addition, the MDA level showed a significantly negative correlation with *Deferribacteres*. Moreover, the results indicated that the GSH content was negatively related to the abundances of *Lactobacillus*, *Dubosiella, Roseburia*, *Lachnospiraceae*_NK4A136_group and *Odoribacter*.

### 3.9. Main Phytochemical Compounds in Different Black and Dark Tea Extracts

**Figure 11:**
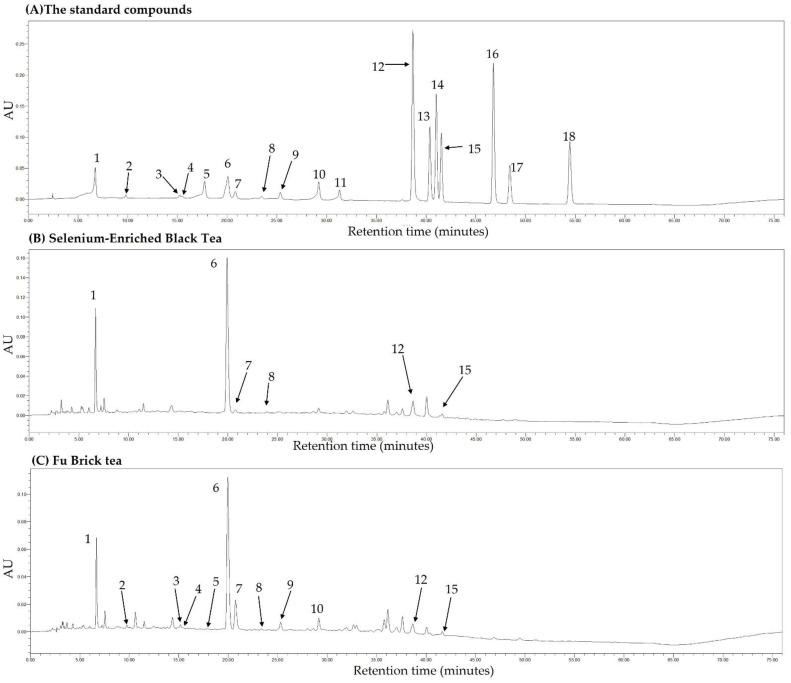
The HPLC chromatograms under 254 nm wavelength of the standard compounds (**A**), selenium-enriched black tea (**B**), and Fu brick tea (**C**). 1, gallic acid; 2, gallocatechin; 3, epigallocatechin; 4, catechin; 5, chlorogenic acid; 6, caffeine; 7, epigallocatechin gallate; 8, epicatechin; 9, gallocatechin gallate; 10, epicatechin gallate; 11, catechin gallate; 12, ellagic acid; 13, myricetin; 14, quercitrin; 15, astragalin; 16, quercetin; 17, theaflavin; 18, kaempferol.

In the present study, the main phytochemical compounds in three deep-fermented black tea extracts and three postfermented dark tea extracts were determined by HPLC, and the detail results of main components in different black and dark tea extracts are displayed in Table 4. Meanwhile, the chromatograms under 254 nm wavelength of the standard components, Yingde black tea (BTea3), and Fu brick tea (DTea1) are shown in Figure 11.

Thirteen ingredients have been determined to be present in different black and dark tea extracts, including eight catechins and five other active substances (gallic acid, chlorogenic acid, caffeine, ellagic acid and astragalus). The 8 catechins are EGCG, gallocatechin gallate (GCG), epicatechin gallate (ECG), catechin gallate (CG), gallocatechin (GC), epigallocatechin (EGC), epicatechin (EC), and catechin. The results indicated that the dark teas contained more abundant catechins compared with the black teas, and that all eight catechins were detected in dark tea. However, the content of catechin in black and dark tea was low compared with green tea [27], because black tea and dark tea are highly fermented [63]. In addition, we found that caffeine was abundant in all black and dark teas, with concentrations ranging from 60.85 ± 0.96 to 96.63 ± 0.58 mg/g. It has been reported that caffeine consumption could promote energy metabolism and is closely associated with improvement in the obesity and NAFLD [64]. Moreover, gallic acid was determined in different black and dark teas ranging from 8.92 ± 0.33 to 77.17 ± 0.94 mg/g, which was associated with the degradation of catechins during the fermentation process of black and dark teas.

## 4. Conclusions

In this study, the protective effects of supplementation with different black teas and dark teas on obesity and NAFLD induced by the long-term HFD exposure and the potential role of gut microbiota in the prevention and management of NAFLD were investigated and explored. The results showed that supplementation with different black and dark tea extracts could significantly suppress the energy intake, alleviate abnormal accumulation of visceral fat, and prevent obesity, hepatic abnormal lipid deposition and liver steatosis in HFD-fed mice at varying degrees. Additionally, the structure and composition of intestinal microbiota communities were changed by the prolonged HFD exposure, which could be shifted by certain teas. For instance, Dianhong tea (BTea2) and Liupao tea (DTea2) interventions could significantly decrease the ratio of *Firmicutes* to *Bacteroidetes,* and selenium-enriched black tea (BTea1) and selenium-enriched dark tea (DTea3) supplementation could remarkably reduce the relative abundance of *Actinobacteria* compared with the model group. Moreover, these teas could partly shift the relative abundances of *Allobaculum*, *Roseburia* and *Dubosiella* at the genus level. Overall, black teas and dark teas could prevent features of obesity and NAFLD induced by HFD in mice, which might be due to the modulation of gut microbiota. Therefore, gut microbiota might be a potential target for the therapeutic strategy development to prevent and manage NAFLD, and these teas have great potential to be used as functional beverages against obesity and NAFLD.

## Figures and Tables

**Table 1:** The detailed information of three black teas and three dark teas.

Number	Name	Category	Fermentation Degree	Production Place
BTea1	Selenium-Enriched Black Tea	Black Tea	Deep-fermented	Enshi, Hubei
BTea2	Dianhong Tea	Black Tea	Deep-fermented	Xishuangbanna, Yunnan
BTea3	Yingde Black Tea	Black Tea	Deep-fermented	Yingde, Guangdong
DTea1	Fu Brick tea	Dark Tea	Postfermented	Changsha, Hunan
DTea2	Liupao Tea	Dark Tea	Postfermented	Wuzhou, Guangxi
DTea3	Selenium-Enriched Dark Tea	Dark Tea	Postfermented	Enshi, Hubei

**Table 2:** Effect of different black and dark teas on gut microbial richness and diversity.

Groups	Chao1	Shannon_2	Simpson
ND	528.2 ± 86.0	4.80 ± 0.50	0.092 ± 0.043
HFD	519.1 ± 88.7	4.60 ± 0.59	0.111 ± 0.044
BTea1	650.5 ± 27.0 ^**^	5.13 ± 0.24	0.105 ± 0.021
BTea2	436.5 ± 94.2 ^aa^	4.03 ± 0.70 ^aa^	0.168 ± 0.100
BTea3	583.1 ± 80.5 ^bb^	4.91 ± 0.62 ^b^	0.089 ± 0.026 ^b^
DTea1	582.0 ± 52.6 ^aaa,bb^	4.63 ± 0.59	0.108 ± 0.044
DTea2	477.6 ± 108.3 ^aa^	4.26 ± 0.65 ^a^	0.149 ± 0.056
DTea3	570.4 ± 80.9 ^b^	4.84 ± 0.67 ^b^	0.108 ± 0.057

Note: The results are expressed as mean ± standard deviation. ND, the control group (normal diet group); HFD, the model group (high-fat diet group); BTea1, selenium-enriched black tea; BTea2, Dianhong tea; BTea3, Yingde black tea; DTea1, Fu brick tea; DTea2, Liupao tea; DTea3, selenium-enriched dark tea. ** *p* < 0.01, the tea extract supplementary groups compared with the HFD group. ^a^ *p* < 0.05, ^aa^ *p* < 0.01, ^aaa^ *p* < 0.001, the other tea extract supplementary groups compared with the BTea1 tea extract supplementary group. ^b^ *p* < 0.05, ^bb^ *p* < 0.01, the other tea extract supplementary groups compared with the BTea2 tea extract supplementary group.

**Table 3:** Effect of different black and dark teas on gut microbial composition.

Group1 vs. Group2	Df	Sums of Sqs	MeanSqs	F.Model	R^2^	Pr(>F)
BTea1 vs. BTea2	1	0.595	0.595	8.93	0.527	0.012
BTea1 vs. BTea3	1	0.264	0.264	7.37	0.48	0.006
BTea1 vs. DTea1	1	0.145	0.145	3.63	0.312	0.015
BTea1 vs. DTea2	1	0.643	0.643	14.8	0.65	0.014
BTea1 vs. DTea3	1	0.425	0.425	6.94	0.464	0.004
BTea1 vs. HFD	1	0.682	0.682	16.0	0.616	0.003
BTea1 vs. ND	1	1.87	1.870	65.0	0.867	0.004
BTea2 vs. BTea3	1	0.318	0.318	3.66	0.314	0.017
BTea2 vs. DTea1	1	0.302	0.302	3.33	0.294	0.035
BTea2 vs. DTea2	1	0.0644	0.0644	0.683	0.0786	0.698
BTea2 vs. DTea3	1	0.0928	0.0928	0.826	0.0936	0.568
BTea2 vs. HFD	1	0.122	0.122	1.46	0.128	0.162
BTea2 vs. ND	1	0.836	0.836	12.0	0.546	0.002
BTea3 vs. DTea1	1	0.0914	0.0914	1.52	0.160	0.151
BTea3 vs. DTea2	1	0.243	0.243	3.82	0.323	0.006
BTea3 vs. DTea3	1	0.155	0.155	1.91	0.192	0.081
BTea3 vs. HFD	1	0.215	0.215	3.66	0.268	0.014
BTea3 vs. ND	1	1.38	1.38	30.7	0.754	0.006
DTea1 vs. DTea2	1	0.317	0.317	4.68	0.369	0.007
DTea1 vs. DTea3	1	0.215	0.215	2.51	0.239	0.036
DTea1 vs. HFD	1	0.326	0.326	5.26	0.345	0.002
DTea1 vs. ND	1	1.48	1.48	30.7	0.754	0.001
DTea2 vs. DTea3	1	0.0634	0.0634	0.712	0.0818	0.611
DTea2 vs. HFD	1	0.0734	0.0734	1.13	0.102	0.371
DTea2 vs. ND	1	0.871	0.871	17.1	0.631	0.002
DTea3 vs. HFD	1	0.0961	0.0961	1.22	0.108	0.294
DTea3 vs. ND	1	0.875	0.875	13.4	0.573	0.005
HFD vs. ND	1	1.13	1.13	23.0	0.657	0.001
All Groups	7	3.66	0.523	8.14	0.613	0.001

Note: The effects of different black and dark teas on the gut microbiota community structure at genus level based on permutational MANOVA (PERMANOVA) analysis using Bray–Curtis distance. ND, the control group (normal diet group); HFD, the model group (high-fat diet group); BTea1, selenium-enriched black tea; BTea2, Dianhong tea; BTea3, Yingde black tea; DTea1, Fu brick tea; DTea2, Liupao tea; DTea3, selenium-enriched dark tea.

**Table 4:** The contents (mg/g) of main phytochemicals in different black and dark teas.

Main Phytochemicals	Selenium-Enriched Black Tea	Dianhong Tea	Yingde Black Tea	Fu Brick Tea	Liupao Tea	Selenium-Enriched Dark Tea
gallic acid	33.28 ± 0.57	23.33 ± 0.92	15.57 ± 0.42	21.75 ± 0.47	8.92 ± 0.33	77.17 ± 0.94
gallocatechin	-	-	-	9.44 ± 0.29	3.33 ± 0.14	21.18 ± 1.74
epigallocatechin	-	-	-	14.49 ± 0.61	5.75 ± 0.07	37.75 ± 0.38
catechin	-	-	11.04 ± 0.24	10.85 ± 0.34	12.30 ± 0.28	8.27 ± 0.03
chlorogenic acid	-	-	2.19 ± 0.03	3.30 ± 0.05	-	-
caffeine	96.63 ± 0.58	95.25 ± 0.90	60.85 ± 0.96	72.92 ± 0.57	92.64 ± 1.07	67.55 ± 0.62
epigallocatechin gallate	9.30 ± 0.55	-	-	59.56 ± 1.94	-	42.85 ± 3.10
epicatechin	5.10 ± 0.07	6.44 ± 0.21	7.39 ± 0.22	8.27 ± 0.28	7.04 ± 0.22	11.87 ± 0.21
gallocatechin gallate	-	-	-	23.55 ± 1.09	19.40 ± 0.73	13.81 ± 0.40
epicatechin gallate	-	5.90 ± 0.14	5.92 ± 0.28	6.86 ± 0.26	-	4.51 ± 0.26
catechin gallate	-	-	-		4.15 ± 0.47	-
ellagic acid	2.76 ± 0.13	3.59 ± 0.21	-	1.68 ± 0.08	3.16 ± 0.10	-
myricetin	-	-	-		-	-
quercetin	-	-	-		-	-
astragalin	7.32 ± 0.41	3.95 ± 0.38	-	1.86 ± 0.01	-	2.27 ± 0.29
quercitrin	-	-	-	-	-	-
theaflavin	-	-	-	-	-	-
kaempferol	-	-	-	-	-	-

Abbreviations: -, mean not detected. The results are expressed as mean ± standard deviation.

## Data Availability

All data presented within the article is available upon reasonable request from the corresponding authors.
